# Vitamin D3-Deficient Diet Promotes Pulmonary Fibrosis Development in Murine Model of Hypersensitivity Pneumonitis

**DOI:** 10.3390/ijms262411770

**Published:** 2025-12-05

**Authors:** Marta Kinga Lemieszek, Michał Chojnacki, Iwona Paśnik, Wiktoria Gawryś, Alicja Wilczyńska, Ilona Leśniowska, Jakub Anisiewicz, Michał Kiełbus

**Affiliations:** 1 Department of Medical Biology, Institute of Rural Health, 20-090 Lublin, Poland; chojnacki.michal@imw.lublin.pl (M.C.);; 2 Department of Biochemistry and Molecular Biology, Medical University of Lublin, 20-059 Lublin, Poland; michal.kielbus@umlub.edu.pl

**Keywords:** vitamin D, cholecalciferol, calcitriol, pulmonary fibrosis, hypersensitivity pneumonitis, extrinsic allergic alveolitis, *Pantoea agglomerans*

## Abstract

Although vitamin D3 (VD3) deficiency has been recognized as a harmful agent in several respiratory diseases, the present study is the first one to investigate its influence on the development of hypersensitivity pneumonitis (HP). This research was conducted in a murine model of HP, wherein pulmonary fibrosis was induced by antigen of *Pantoea agglomerans*. VD3 deficiency was provoked by diet with 10-times less cholecalciferol than feed given to VD3-sufficient mice. Before and after 14 and 28 days of nebulization, lung function was evaluated. Moreover, at indicated time points, lungs were collected and subjected to histological assessment, flow cytometry, gene expression assays, and ELISA. The performed research showed a higher sensitivity of VD3-deficient mice to fibrosis response to *P. agglomerans* antigen, which was strongly associated with enhanced epithelial-to-mesenchymal transition, the signs of which were over-expression of EMT-transcription factors (*Snail2*, *Zeb1*, *Zeb2*) and mesenchymal cell markers (*Cdh2*/N-cadherin, *Acta2*/SMA, *Fn1*/Fibronectin, *Vim*/Vimentin). Indicated negative changes in VD3-deficient mice with developed HP were supported by deepening calcitriol deficiency and worsening respiratory functions, including the frequency of breathing, minute volume, total cycle times, expiratory and inspiratory time. Moreover, typical for VD3-deficient mice with HP, there was also an increased influx of immune cells into the lungs (especially neutrophils, macrophages, dendritic cells and lymphocytes Tc), a disturbed cytokine profile with over-production of growth factors favoring fibrosis (FGF2 and TGFβ), and lowered synthesis of several cytokines (IL1β, IL6, IL12, IL4 IL10, IL13). The present study reveals that VD3 deficiency promotes the development of pulmonary fibrosis in the murine model of HP.

## 1. Introduction

The term vitamin D includes vitamin D2 (ergocalciferol) and vitamin D3 (cholecalciferol). Ergocalciferol is found in mushrooms and plants, while cholecalciferol is present in animals. Thus, depending on food origin, mammals consume ergocalciferol or cholecalciferol. Nevertheless, the most important source of vitamin D3 is skin synthesis in response to UVB radiation. Despite the form, both ergocalciferol and cholecalciferol undergo the same two-step activation to 25(OH)-vitamin D3 (calcidiol), with the participation of liver enzymes (25-hydroxylases CYP2R1 and CYP27A1) and further to bioactive 1,25(OH)_2_-vitamin D3 (calcitriol), with the involvement of enzymes (1-hydroxylase CYP27B1) mostly present in kidneys [1,2]. It should be emphasized that 25(OH)-vitamin D3 is the dominant circulating form of vitamin D3 which, thanks to a long half-life (2–3 weeks), has been used to determine vitamin D3 levels. Instead, 1,25(OH)_2_-vitamin D3, recently indicated as a hormone regulating a wide range of biological processes in the body, is rarely determined because of its short half-life (4–6 h) [3,4,5].

Although humans are able to obtain vitamin D3 from two independent sources (endogenous synthesis and diet), its deficiency, defined as calcidiol serum concentration below 20 ng/mL or 50 nmol/L was recognized as a pandemic with serious health consequences [6]. Research strictly dedicated to the respiratory system revealed that a shortage of vitamin D3 impaired pulmonary function and increased the risk of development and severe course of asthma, cystic fibrosis, chronic obstructive pulmonary disease (COPD), lung cancer, allergic inflammations, and respiratory infections, as well as idiopathic pulmonary fibrosis (IPF) [7,8,9,10,11,12]. Unfortunately, there is a lack of research investigating the impact of vitamin D3 deficiency on the development and progression of pulmonary fibrosis related to hypersensitivity pneumonitis, which is estimated to be one of the most frequent reasons for pulmonary fibrosis worldwide [13,14]. The present study is an attempt to shed light on this occurrence, as well as to verify the possibility of using cholecalciferol supplementation in the prevention of the indicated disease.

Hypersensitivity pneumonitis (HP) represents an interstitial lung disease in which chronic exposure to a diverse range of airborne antigens may induce a hypersensitivity immune reaction in the respiratory tract of susceptible individuals, which in extreme cases leads to fibrosis [15,16]. HP comprises a number of variants, e.g., farmer’s lung, malt fever, pigeon breeder’s lung, bird fancier’s lung, mushroom worker’s lung, wood fibre alveolitis, soya dust alveolitis or suberosis, which show a similar clinical course, but are caused by different types of antigens, including bacteria, fungi, mycobacteria, plant and animal proteins, chemicals, and metals [15,17]. Because of the great variety and distribution of HP-triggered antigens, millions of individuals are exposed to them as part of their occupational, home, or recreational environments, which translates into the disease incidence [13,14]. However, the indicated multitude of HP varieties obscures the scale of the problem and partly explains why prevention and treatment of this disease are still a challenge for contemporary medicine. We take up this challenge focused on the inhibition of HP development, leading to lung fibrosis, through a diet with an optimal amount of vitamin D3.

Our previous studies in C57BL/6 mice with both diet-induced vitamin D3 deficiency (VD3-deficient mice) and HP induced by long-term inhalations with *Pantoea agglomerans* antigen (HP—etiological factor) revealed a beneficial effect of calcidiol and calcitriol inhalation on the disease course. Vitamin D3 metabolites administered at doses restoring physiological pulmonary levels of 1,25(OH)_2_D3 in VD3-deficient mice with HP inhibited fibrosis development by limiting the excessive influx of immune cells into the lung parenchyma and reducing the over-production of factors promoting inflammation and pathological tissue repair [18]. Further studies have shown that the discovered favourable influence of inhaled vitamin D3 metabolites was also associated with inhibition of epithelial-mesenchymal transition (EMT), a phenomenon underlying fibrosis, by limiting the expression of EMT transcription factors and mesenchymal cell markers, as well as enhancing the expression of epithelial cell markers [19]. A limitation of these studies was their omission of the impact of vitamin D3 deficiency itself on the HP course. Consequently, we do not know the extent to which individual harmful factors (vitamin D3 deficiency and *P. agglomerans* antigen) were responsible for the observed pathological changes. This study aims to clarify this issue. Furthermore, comparing data from animal models of HP under conditions of vitamin D3 deficiency and optimal vitamin D3 intake will allow us to determine whether maintaining physiological calcitriol concentrations through an appropriate diet will inhibit or prevent the development of the disease.

## 2. Results

### 2.1. The Amount of Cholecalciferol in Animals’ Diets as Well as the Developed HP Impact on Pulmonary and Serum Levels of Calcitriol

The evaluation of endogenous serum and pulmonary concentration of calcitriol in VD3-deficient and VD3-sufficient mice shows a statistically significantly lower amount of this biologically active form of vitamin D3 in animals on a cholecalciferol-reduced diet. The collected data are presented in Table 1. The pulmonary level of calcitriol decreased from 30.21 pg/mL (VD3S mice) to 18.21 pg/mL (VD3D mice); similarly, its serum level lowered from 132.71 pg/mL(VD3S mice) to 98.63 pg/mL (VD3D mice). The amount of calcitriol was also affected by animals’ exposure to *P. agglomerans* antigen, and the observed reduction of this metabolite was more evident in animals with vitamin D3 deprivation. The pulmonary level of calcitriol after treatment with antigen decreased from 27.89 pg/mL (VD3S PA 14 days) to 24.07 pg/mL (VD3S PA 28 days), and from 15.85 pg/mL (VD3D PA 14 days) to 13.30 pg/mL (VD3D PA 28 days). A similar pattern of changes was observed in VD3D mice, whereas 14 and 28 days of exposure to *P. agglomerans* caused an additional reduction of calcitriol from 78.68 pg/mL to 69.86 pg/mL, respectively. In the case of mice on a standard diet, the serum level of calcitriol in antigen-treated mice was quite similar at both investigated time points: 116.73 pg/mL (14 days) and 119.34 pg/mL (28 days).

### 2.2. VD3 Deficiency Worsens the Respiratory Functions During HP Development

**Figure 1:**
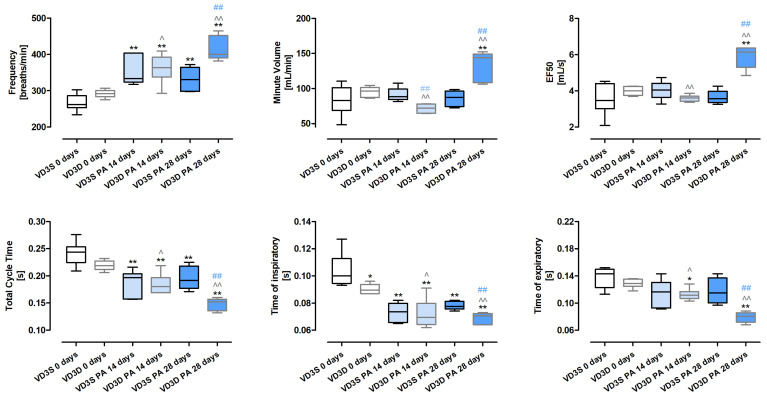
Alterations in pulmonary function caused by vitamin D3 deficiency and progressive hypersensitivity pneumonitis. Lung functions were investigated using whole-body plethysmography. The investigations were performed at the end of each experiment. Data were collected every 2 s for 10 min. Statistical comparisons are shown for: * (vs. main control, VD3S 0 days); ^ (VD3D PA 14/28 days vs. VD3D 0 days); and # (VD3D vs. VD3S at 14 or 28 days). The number of symbols indicates the significance level: one (*p* < 0.05), two (*p* < 0.01). Numerical data are listed in the Supplementary Table S1.

Whole-body plethysmography (Figure 1 and Table S1) revealed that only Time of inspiratory (Ti) was significantly affected by the cholecalciferol-deprived diet. Ti in untreated mice decreased in response to cholecalciferol deficiencies from 0.10 s to 0.09 s.

The negative impact of VD3-deficiencies on animals’ lung function showed up during HP development and intensified in a time-dependent manner. The frequencies of breathing increased after 14 and 28 days of treatment with *P. agglomerans* antigen from 291.75 breaths/min to 363.22 breaths/min and 399.64 breaths/min, respectively. On the contrary, under the indicated conditions, Total Cycle Time (TCT) dropped from 0.22 s to 0.18 s to 0.15 s; similarly, Time of expiratory (Te) reached the following values: 0.13 s, 0.11 s and 0.08 s, while Ti in both the 14th and 28th days of treatment remained at the level of 0.07 s. A different pattern of changes than previously presented was observed for MV (Minute Volume) and EF50 (mid-tidal expiratory flow), which decreased after 14 days of VD3-deficient mice inhalation with antigen and then significantly increased. Alterations in the indicated parameters were presented as follows: MV 96.18 mL/min (VD3D 0 days), 71.99 mL/min (VD3D 14 days), 143.81 mL/min (VD3D 28 days); EF50 3.99 mL/s (VD3D 0 days), 5.61 mL/s (VD3D 14 days), 6.15 mL/s (VD3D 28 days).

VD3-sufficient mice respond to *P. agglomerans* antigen in a slightly different way than animals with vitamin D3 deprivation. Foremost, MV, EF50, and Te were not affected by antigen inhalations. Significantly, Ti and TCT decreased in response to *P. agglomerans* antigen, but only TCT changed in a time-dependent manner (Ti: 0.1 s, 0.07 s, 0.08 s; TCT: 0.24 s, 0.2 s, 0.19 s). Frequency of breathing also worsened during *P. agglomerans* treatment, but changes recorded after 14 and 28 days of inhalation were on similar levels (F: 261.87 breaths/min, 333.89 breaths/min, 331.04 breaths/min).

Comparison data collected from HP mice on different diets revealed that VD3-deficiency distinctly worsens respiratory functions during disease development. All investigated parameters (F, MV, EF50, TCT, Ti, Te) in VD3-deficient mice were much more disturbed by 28 days of exposure to *P. agglomerans* antigen than in VD3-sufficient animals.

### 2.3. VD3 Deficiency Intensifies Inflammation and Promotes Pulmonary Fibrosis in Response to Inhalations with HP Inducers

**Figure 2:**
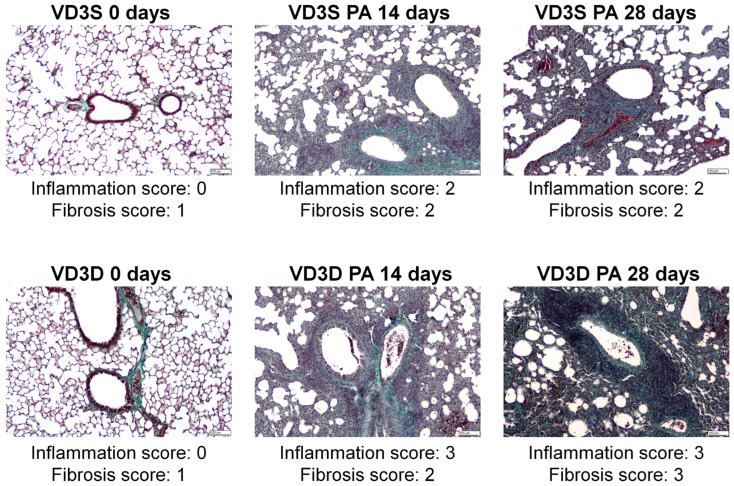
Changes in lung tissue morphology caused by vitamin D3 deficiency and progressive hypersensitivity pneumonitis. Lungs obtained from untreated and antigen-treated mice on standard or cholecalciferol-restricted diet were subjected to Masson trichrome staining and assessed under light microscopy at 100× magnification. Representative photographs of lung sections are shown. Visual quantification of inflammation and fibrosis scores (five-stage system, 0–4) as acquired by a pathologist is presented.

As presented in Figure 2, the examination of lung sections showed discrete changes (focally thickened alveolar septa) in the tissue morphology of untreated VD3-deficient mice. On the contrary, 14 days of inhalation with *P. agglomerans* antigen induced significant changes in both VD3-sufficient and VD3-deficient mice. Indicated research groups were characterized by pulmonary oedema, significant centrolobe and interstitial inflammation (clear influx of granulocytes, mostly neutrophils and reactive pneumocytes, were observed), and alveolar distortion and thickening of their walls. Median lung injury scores in VD3-sufficient and VD3-deficient mice were as follows: 2 and 3 in the case of inflammatory response, and 2 and 2 in the case of fibrosis. Chronic exposure to *P. agglomerans* antigen (28 days) did not cause additional changes in the lung tissue of mice on a standard diet (median scores for inflammation and fibrosis maintained on level 2), while significantly intensifying signs of fibrosis in VD3-deficient mice led to obstruction of the alveoli lumen (median score for inflammation was 2, while median score for fibrosis increased to 3). It should be emphasized that VD3-deficient mice revealed a stronger inflammatory response than VD3-sufficient mice at both investigated time points, while the fibrotic reaction was significantly higher in VD3-deficient mice only after chronic exposure to *P. agglomerans* antigen.

### 2.4. VD3 Deficiency Increases the Influx of Immune Cells into the Lungs in Response to Chronic Exposure to P. agglomerans Antigen

**Figure 3:**
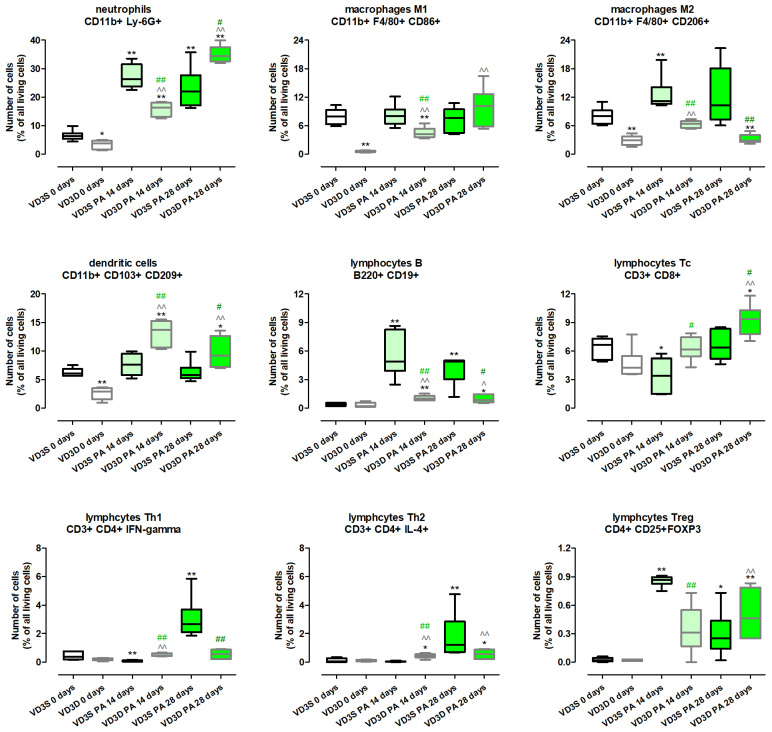
Changes in pulmonary composition of immune cells in response to vitamin D3 deficiency and progressive hypersensitivity pneumonitis. Lung tissue cells labelled with antibodies recognized the selected immune cells were detected using flow cytometry. A minimum of 100,000 cells were acquired and examined. Data are presented as percentages of all living cells. Statistical comparisons are shown for * (vs. main control, VD3S 0 days); ^ (VD3D PA 14/28 days vs. VD3D 0 days); and # (VD3D vs. VD3S at 14 or 28 days). The number of symbols indicates the significance level: one (*p* < 0.05), two (*p* < 0.01). Numerical data are listed in the Supplementary Table S2.

Figure 3 and Table S2 present the results of examination of immune cell composition and show that VD3 diet deprivation, compared to a standard diet, significantly lowered the amount of neutrophils (decrease from 6.27% to 3.71%), dendritic cells (decrease from 6.04% to 2.90%), M1 macrophages (decrease from 7.93% to 0.52%), and M2 macrophages (decrease from 8.01% to 2.88%). On the contrary, lymphocytes B, Tc, Treg, Th1 and Th2 were not affected by cholecalciferol restriction.

VD3-deficient mice inhalation with *P. agglomerans* antigen increased the level of most of the investigated immune cells, the amount of which was even higher than noted in untreated VD3-sufficient mice. Indicated changes were recorded at both time points in the case of neutrophils (16.37% and 34.47%) dendritic cells (13.72% and 9.2%), lymphocytes B (1.01% and 0.86%), lymphocytes Th2 (0.46% and 0.56%), and M1 macrophages (4.23% and 10.09%). Increased immune cell influx in VD3-deficient mice in response to inhalations with *P. agglomerans* antigen was also recorded in the case of M2 macrophages and lymphocytes Th1 after 14 days of treatment, while elevated levels of lymphocytes Tc and Treg were observed on the 28th day of inhalations. Among indicated cell subpopulations, the strongest increase in quantity was noted in lymphocytes Tc and Treg, which reached main control levels after 14 days of *P. agglomerans* treatment (Tc: 6.14%; Treg: 0.31%), then clearly crossed that line after 28 days of inhalations (Tc: 9.36%; Treg: 0.46%). On the contrary, the weakest alterations in M2 macrophages representation were noted; their amounts at both investigated time points were significantly lower than the main control level (6.41% and 2.79%).

Immune cell response of VD3-sufficient mice to inhalations with *P. agglomerans* antigen revealed a more diverse range of reactions. The amounts of dendritic cells and M1 macrophages did not change during HP development; similarly, the number of lymphocytes Th2 after 14 days of treatment, and lymphocytes Tc as well as M2 macrophages after 28 days of treatment, were maintained at the main control level. On the contrary, VD3-sufficient mice inhalation with antigen significantly increased the amount of neutrophils (26.34% and 21.97%), B lymphocytes (4.88% and 4.89%), and Treg lymphocytes (0.86% and 0.25%) at both investigated time points, while an increase in M2 macrophage (11.19%) infiltration was observed only on the 14th day of the nebulization, and elevated quantities of Th1 (2.67%) and Th2 (1.21%) lymphocytes were recorded only at the end of the study. Opposite to changes observed in VD3-deficient mice, animals on the standard diet exposed to *P. agglomerans* antigen for 14 days revealed inhibition of Tc lymphocytes (3.38% vs. 6.64%) and Th1 lymphocytes (0.07% vs. 0.36%) influxes to pulmonary compartments compared to untreated mice.

The comparison data collected from VD3-sufficient and VD3-deficient animals with HP showed that during disease development, changes of a similar nature were observed in subpopulations of neutrophils, lymphocytes B, M2 macrophages at both examined time points, while the pattern of changes in lymphocytes Th2 and Treg was similar in animals inhaled with antigen of *P. agglomerans* for 28 days. On the contrary, vitamin D3 status impacted the response of other investigated immune cells. Dendritic cells, M1 macrophages (both time points), lymphocytes Th2 (14th day), and lymphocytes Tc (28th day), the level of which did not change in VD3-sufficient mice with HP, significantly accumulated in the lungs of VD3-deficient mice treated with antigen. Dependent on VD3 status, differences in immune cell composition were the clearest in the case of lymphocytes Tc and lymphocytes Th1, which significantly decreased in VD3-sufficient mice after 14 days of *P. agglomerans* treatment, while under the same conditions in animals on a VD3-deficient diet, cells were elevated (Th1) or not affected (Tc).

### 2.5. VD3 Deficiency Disturbs the Cytokine Profile of HP Mice, Significantly Reducing the Amount of Pro-Inflammatory and Anti-Inflammatory Cytokines

**Figure 4:**
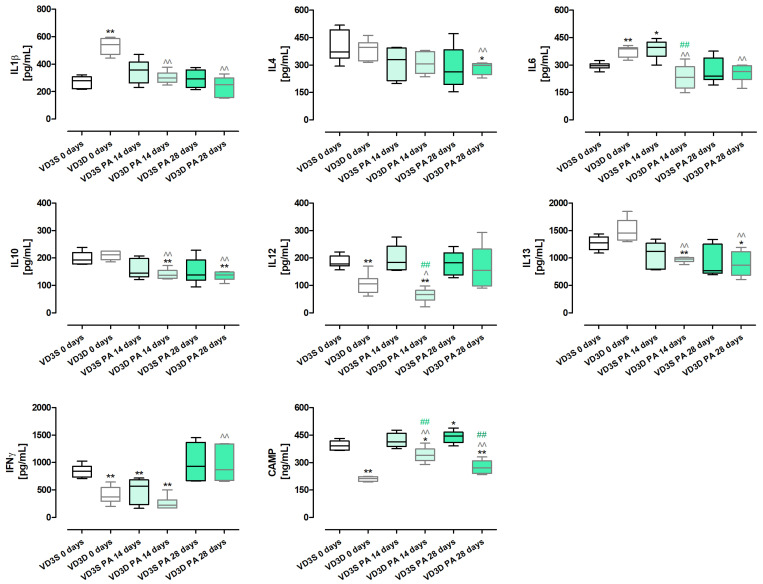
Alterations in the concentrations of selected cytokines and CAMP caused by vitamin D3 deficiency and progressive hypersensitivity pneumonitis. Protein concentration was determined in lung homogenates using dedicated ELISA kits. Statistical comparisons are shown for * (vs. main control, VD3S 0 days); ^ (VD3D PA 14/28 days vs. VD3D 0 days); and # (VD3D vs. VD3S at 14 or 28 days). The number of symbols indicates the significance level: one (*p* < 0.05), two (*p* < 0.01). Numerical data are listed in the Supplementary Table S3.

As presented in Figure 4 and Table S3, vitamin D3 deprivation significantly lowered the concentrations of IL12 (decrease from 177.96 pg/mL to 105.46 pg/mL), IFNγ (decrease from 840.42 pg/mL to 372.47 pg/mL) and Cathelicidin antimicrobial peptide - CAMP (decrease from 391.51 ng/mL to 211.27 ng/mL). At the same time, it increased the amounts of IL1β (increase from 279.16 pg/mL to 542.45 pg/mL) and IL6 (increase from 296.26 pg/mL to 387.18 pg/mL). The amounts of IL4, IL10, and IL13 were not affected by vitamin D3 status.

VD3-deficient mice nebulization with *P. agglomerans* decreased the amount of most investigated proteins compared to untreated mice on the same diet. Nevertheless, four different patterns of changes were observed: (1) time-dependent decrease: IL1β (300.65 pg/mL and 250.88 pg/mL), IL13 (982.76 pg/mL and 868.15 pg/mL); (2) decrease with the strongest effect on day 14th: IL6 (231.16 pg/mL and 263.67 pg/mL), IL10 (136.95 pg/mL and 137.51 pg/mL); (3) decrease after 14 days of treatment: IL12 (66.4 pg/mL); (4) decrease after 28 days of treatment: IFNγ (868.71 pg/mL). Changes of a different nature were observed in the case of immune peptide CAMP, which, in response to 14 and 28 days of antigen inhalations, was significantly elevated, reaching 339.57 ng/mL and 271.28 ng/mL, respectively. Similarly, the amount of IFNγ was not affected by 14 days of mice exposure to antigen but significantly increased at the end of the study, reaching 868.71 pg/mL.

The cytokine profile in VD3-sufficient mice was resistant to changes induced by chronic exposure to *P. agglomerans* antigen. Among the examined cytokines, significant changes were noted only in the case of IL6, IFNγ, and CAMP. IL6 increased from 296.26 pg/mL (main control) to 396.1 pg/mL after 14 days of treatment. In the same compared research groups, an opposite effect was recorded in the case of IFNγ, the concentration of which decreased from 840.42 pg/mL to 565.27 pg/mL. On the contrary, the amount of CAMP significantly increased at the end of the study, reaching 444.65 ng/mL.

Comparison data collected from VD3-sufficient and VD3-deficient mice with HP showed significant changes in the expression of IL6, IL12 on the 14th day of the experiment, as well as CAMP at both investigated time points. Concentrations of the above-mentioned cytokines were significantly lower in VD3-deficient mice than in mice on a vitamin D3-sufficient diet, indicating a relationship between their levels and vitamin D3 status. Simultaneously, the lack of significant changes in the concentration of other proteins in VD3-sufficient and VD3-deficient mice in response to antigen exposure, may suggest that their expression did not depend on vitamin D3 status.

### 2.6. VD3 Deficiency Increases the Expression of Fibrosis Markers and Factors, Promoting This Pathology in Response to HP Inducer

**Figure 5:**
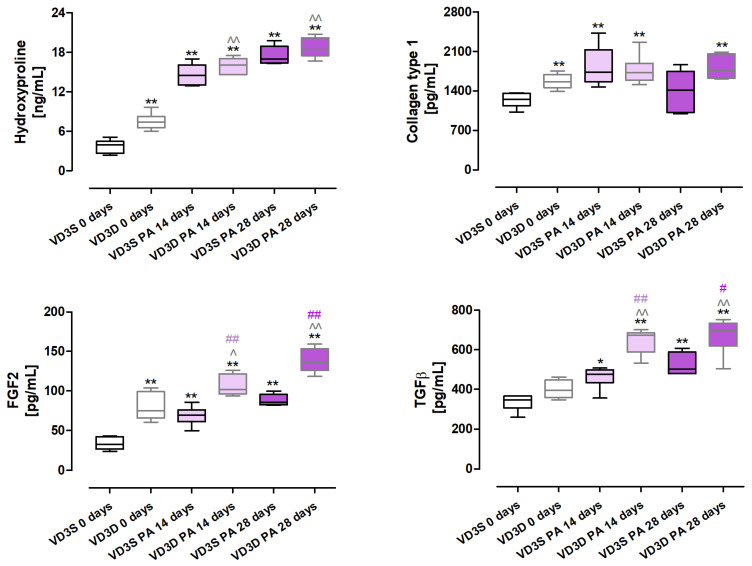
Changes in extracellular matrix deposition in response to vitamin D3 deficiency and progressive hypersensitivity pneumonitis. Protein concentrations in lung homogenates were examined using ELISA kits. Statistical comparisons are shown for * (vs. main control, VD3S 0 days); ^ (VD3D PA 14/28 days vs. VD3D 0 days); and # (VD3D vs. VD3S at 14 or 28 days). The number of symbols indicates the significance level: one (*p* < 0.05), two (*p* < 0.01). Numerical data are listed in the Supplementary Table S4.

As presented in Figure 5 and Table S4, cholecalciferol restriction significantly increased the deposition of extracellular matrix components, the signs of which were the following changes in protein concentrations: hydroxyproline (increase from 3.92 ng/mL to 7.4 ng/mL), collagen type I (increase from 1252.67 pg/mL to 1561.80 pg/mL). Moreover, concentrations of FGF2 (Fibroblast Growth Factor 2) also increased from 32.63 pg/mL to 74.67 pg/mL in response to cholecalciferol restriction in animals’ diets.

Changes in the expression of hydroxyproline, FGF2, and TGFβ escalated in a time-dependent manner in VD3-deficient mice in response to *P. agglomerans* antigen; concentrations of indicated molecules after 14 and 28 days of nebulization were as follows: hydroxyproline, 16.09 ng/mL and 18.43 ng/mL; FGF2, 101.83 pg/mL and 135.74 pg/mL; and TGFβ, 674.64 pg/mL and 695.23 pg/mL. Instead, the amount of collagen type 1 did not change in VD3-deficient mice during HP development; however, its over-expression in untreated mice on the same diet indicated the dependence of its level on VD3 status.

VD3-sufficient mice exposed to *P. agglomerans* antigen also significantly elevated the concentration of three out of four proteins which, after 14 and 28 days of treatment, reached the following values: hydroxyproline, 14.49 ng/mL and 16.99 ng/mL; FGF2, 147.12 pg/mL and 212.66 pg/mL; TGFβ, 475.29 pg/mL and 502.78 pg/mL. In the case of type 1 collagen, a significant increase in its concentration was noted only after 14 days of exposure, wherein this protein concentration reached 1733.79 pg/mL.

Analysis of mice response to HP development revealed that VD3 deficiency intensified the extracellular matrix deposition compared to mice on a standard diet, and the observed effect was associated with elevated expression of TGFβ and FGF2.

### 2.7. VD3-Deficiency Promotes EMT Underlying Pulmonary Fibrosis Development in HP

**Figure 6:**
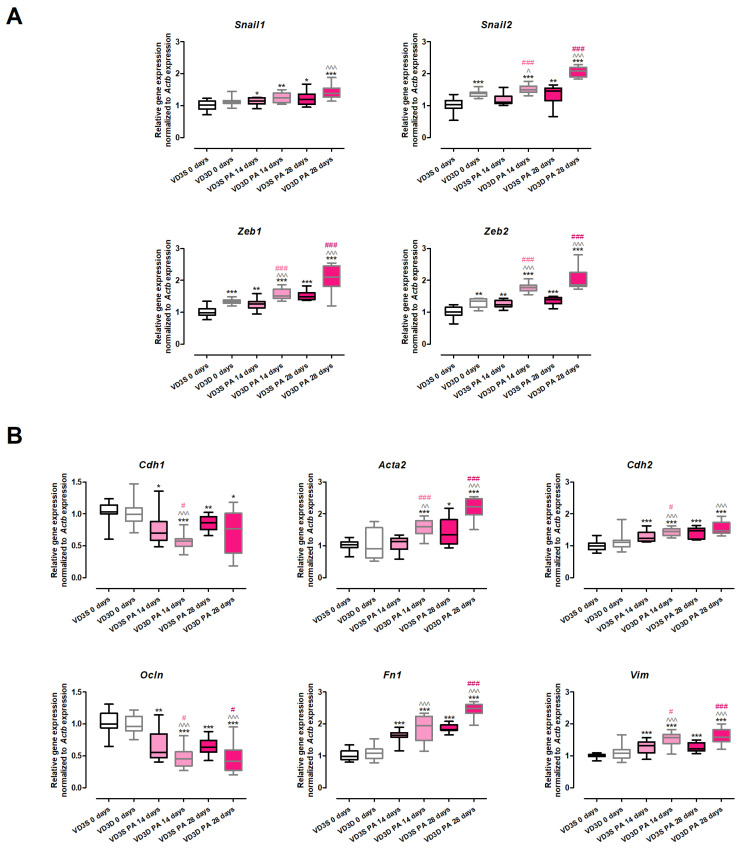
Changes in the gene expression of EMT markers in response to vitamin D3 deficiency and progressive hypersensitivity pneumonitis. Gene expression in lung homogenates was examined by real-time PCR. (**A**) Alterations in the expression of EMT transcription factors. (**B**) Alterations in the transcription of epithelial and mesenchymal cell markers. (**A**,**B**) present relative gene expression. Statistical comparisons are shown for * (vs. main control, VD3S 0 days); ^ (VD3D PA 14/28 days vs. VD3D 0 days); and # (VD3D vs. VD3S at 14 or 28 days). The number of symbols indicates the significance level: one (*p* < 0.05), two (*p* < 0.01), or three (*p* < 0.001). Numerical data are listed in the Supplementary Table S5.

The results of the gene expression assay (Figure 6 and Table S5) clearly demonstrated that VD3-deficiencies significantly up-regulated the expression of the main transcription factors involved in EMT (*Snail2*: 1.03 vs. 1.39; *Zeb1*: 0.99 vs. 1.34; *Zeb2*: 1.02 vs. 1.35). On the contrary, the expression of *Snail1* and the key EMT markers (*Cdh1*, *Ocln*, *Cdh2*, *Acta2*, *Fn1*, *Vim*) was not affected by cholecalciferol deprivation in the animals’ diet.

VD3-deficient mice treated with *P. agglomerans* antigen altered the expression of all investigated genes, causing an increase in the expression of EMT transcription factors and mesenchymal cell markers, as well as down-regulating the expression of epithelial cell markers. Elevated levels of mesenchymal cell markers were noted after 14 and 28 days of mice treatment with the antigen (*Acta2*: 1.60 and 2.23; *Cdh2*: 1.45 and 1.48; *Fn1*: 1.94 and 2.49; *Vim*: 1.57 and 1.59). Similarly, a time-dependent increase in mRNA levels during HP development was recorded in the cases of *Snail2* (1.51 and 2.08), *Zeb1* (1.51 and 2.10), and *Zeb2* (1.77 and 1.86). On the contrary, a significant increase in the expression of *Snail1* (1.39) was recorded only after 28 days of VD3-deficient mice nebulization with *P. agglomerans* antigen. Short (14 days) exposure of VD3-deficient mice to the antigen lowered the expression of both investigated epithelial cell markers (*Cdh1* decreased from 1.00 to 0.57; *Ocln* decreased from 0.96 to 0.45), while this alteration pattern was maintained at the end of the study only in the case of *Ocln* (0.42).

VD3-sufficient mice that inhaled *P. agglomerans* antigen revealed a quite similar pattern of changes in the expression of EMT markers, the signs of which were up-regulation of gene expression of EMT transcription factors and mesenchymal cell markers with simultaneous down-regulation of epithelial cell markers. Among the twelve investigated genes, the expressions of ten were altered at both investigated time-points, while the mRNA levels of two genes (*Acta2* and *Snail2*) were affected only in response to chronic antigen exposure. The gene expressions of the indicated molecules were as follows: (A) epithelial cell markers (*Cdh1*: 0.7 and 0.86; *Ocln*: 0.55 and 0.64); (B) mesenchymal cell markers (*Acta2*: 1.35; *Cdh2*: 1.23 and 1.47; *Fn1*: 1.64 and 1.82; *Vim*: 1.32 and 1.22); (C) transcription factors (*Snail1*: 1.14 and 1.21; *Snail2*: 1.46; *Zeb1*: 1.26 and 1.49; *Zeb2*: 1.24 and 1.40).

The comparison of VD3-deficient and VD3-sufficient mice’s response to inhalations with antigen of *P. agglomerans* demonstrated that cholecalciferol deprivation significantly increased the range of gene alterations. The transcription of epithelial markers was generally lower in VD3-deficient mice than in animals on a regular diet. At the same time, mRNA levels of mesenchymal markers and investigated transcription factors were significantly higher in the case of VD3-deficient mice than in VD3-sufficient ones. It should be emphasized that expression of *Snail1* (both time points), *Fn1* (14th day), *Cdh1* (28th day), and *Cdh2* (28th day) were on similar levels on both types of investigated diets.

### 2.8. VD3 Deficiency Enhanced Mesenchymal Phenotypes Associated with HP-Related Pulmonary Fibrosis

**Figure 7:**
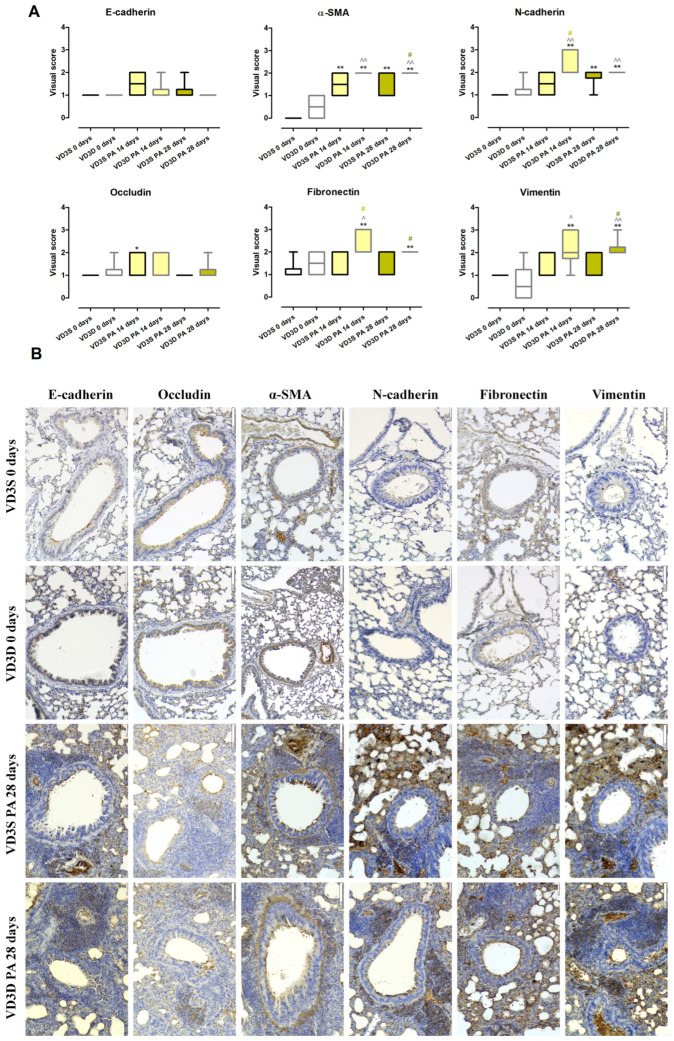
Alterations in the expression of EMT markers in response to vitamin D3 deficiency and progressive hypersensitivity pneumonitis. (**A**) Visual quantification of protein expression on immunohistochemically stained murine lung tissue sections. The visual scores (five-stage system, 0–4) as acquired by a pathologist are presented. Statistical comparisons are shown for * (vs. main control, VD3S 0 days); ^ (VD3D PA 14/28 days vs. VD3D 0 days); and # (VD3D vs. VD3S at 14 or 28 days). The number of symbols indicates the significance level: one (*p* < 0.05), two (*p* < 0.01). Numerical data are listed in the Supplementary Table S6. (**B**) Representative photographs of lung sections stained with antibodies specific for EMT markers captured under a light microscope at 100× magnification.

As presented in Figure 7A and Table S6, histopathologist examination of lung sections stained with antibodies specific for EMT markers showed a lack of changes in the expression of E-cadherin, Occludin, N-cadherin, Fibronectin, α-SMA (Alpha-Smooth Muscle Actin), and Vimentin between VD3-deficient and VD3-sufficient animals.

VD3-deficient mice inhalation with *P. agglomerans* antigen did not impact the expression of epithelial markers (E-cadherin and Occludin), but enhanced expression of mesenchymal markers (over-expression at both time points: N-cadherin, α-SMA, and Vimentin; over-expression only after 14 days of treatment: Fibronectin) was noted. Visual scores assigned by a pathologist for EMT markers examined after 14 and 28 days of antigen treatment were as follows: N-cadherin (3 and 2), Fibronectin (2 and 2), α-SMA (2 and 2), and Vimentin (2 and 2).

VD3-sufficient mice were more resistant to EMT induction by *P. agglomerans* antigen, the sign of which was a lack of disorders in the expression of three out of six tested molecules (E-cadherin, Fibronectin and Vimentin). On the contrary, expression of Vimentin increased from 1 to 2 at both investigated time points, while N-cadherin was up-regulated only at the end of the study (visual score 2 on the 28th day of treatment). Over-expression was also observed in the case of Occludin, which reached a visual score of 2 after 14 days of PA inhalations.

Comparison data collected from mice with developed HP revealed dependence on VD3 status disorders in the expression of mesenchymal markers. VD3-deficient mice showed a significantly higher than VD3-sufficient animals’ expression of N-cadherin (3 vs. 1.5) and Fibronectin (2 vs. 1) after 14 days of antigen exposure, as well as over-expressed amounts of α-SMA (2 vs. 1) and Vimentin (2 vs. 1).

Representative photographs of lung sections stained with antibodies specific for EMT markers collected from untreated as well as *P.agglomerans*-treated animals for 28 days are presented in Figure 7B.

## 3. Discussion

Because 80% of vitamin D3 in the human body comes from endogenous synthesis, while 20% comes from diet [6,11,20], the easiest way to obtain the correct level of this metabolite seems to be exposure to sunlight. However, considering the multitude of factors disrupting the process of cholecalciferol synthesis (e.g., age, skin pigmentation, intensity and time of solar radiation, use of sunscreen, and solar filters) [6,8,10,11,21], as well as increasing concerns about the carcinogenic and pro-aging impact of UVB radiation [11,22,23], a better option seems to be vitamin D supplementation. Indeed, most of the strategies for neutralizing vitamin D3 deficiencies, including the prevention and treatment of pulmonary diseases, are based on vitamin D delivery through the digestive route [6,8,11,21,24]. Nevertheless, there is a lack of scientific data (clinical reports as well as in vivo studies) investigating the influence of vitamin D3 food on the development of pulmonary fibrosis in the course of hypersensitivity pneumonitis. Thus, the present study is the first one designed to fill this knowledge gap as well as verify the possibility of using cholecalciferol supplementation in the prevention of the indicated diseases.

The study was conducted in the murine model of HP, wherein pulmonary fibrosis was provoked by *P. agglomerans* antigen administered to C57BL/6 mice by inhalation [25,26]. Animals used in the study were on a diet with a recommended amount of cholecalciferol (0.5 IU/g; VD3-sufficient mice) or on a diet with a reduced amount of cholecalciferol (0.05 IU/g; VD3-deficient mice). A two-week period preceding the actual experiments, during which mice were on a diet with a standard or reduced cholecalciferol content to induce VD3 sufficiency and VD3 deficiency, respectively, corresponded to one year in humans [27]. This period is therefore sufficiently long to induce and then observe the health consequences associated with both VD3 sufficiency and VD3-deficiency. Induced by diet, VD3-deficiency was confirmed by the lowered amount of calcitriol in both serum and lung tissue homogenates. Moreover, calcitriol concentration was also examined in other research groups in order to check how the cholecalciferol amount in animal food, as well as exposure to *P. agglomerans* antigen, impacts VD3 status. The performed study revealed that both indicated factors significantly impacted the serum and pulmonary levels of calcitriol. In all research groups receiving the diet low in cholecalciferol, calcitriol concentrations were significantly lower than those recorded in animals fed a diet rich in this metabolite, both control animals and those exposed to the antigen. It was also observed that exposure to the HP-inducing factor further reduced calcitriol levels in both investigated compartments. While we expected the above-mentioned results, the rate of change observed in VD3-deficient vs. VD3-sufficient mice was quite surprising. In the case of the lung tissue, calcitriol levels decreased 1.2- and 1.4-fold faster on days 14 and 28 of inhalation, respectively, while calcitriol serum concentrations decreased 2.1- and 2.5-fold faster at the indicated time points. The obtained results show for the first time that developed HP significantly reduces the pulmonary and serum amount of calcitriol. Furthermore, collected data clearly demonstrated that the baseline level of calcitriol significantly influenced its concentration recorded in animals with developing HP: higher calcitriol levels (reflecting the amount of cholecalciferol supplied in the diet) in healthy animals reduced their sensitivity to further depletion of the endogenous pool of this metabolite in response to chronic exposure to the HP-inducing antigen.

Since the concentration of calcitriol, responsible for the biological effect of vitamin D3, is strongly dependent on the cholecalciferol amount in the diet as well as exposure to the *P. agglomerans* antigen (HP-inducer), in the next step of the study, we focused on discovering and describing the differences in the animals’ response to VD3-deficiency under physiological and pathological conditions.

Whole-body plethysmography has shown that VD3-deficient mice have significantly shorter Time of inspiration than untreated animals on diets with standard amounts of cholecalciferol. The observed worsening of respiratory function in untreated VD3-deficient mice corresponded with unique research conducted by Zosky et al., who were the first to reveal that a VD3-deprived diet exacerbated lung function by reduction of lung volume. Unfortunately, this observation originated from offspring born to BALB/c mothers, with diet-induced VD3 deficiency [28], but in addition to our previous reports [18,19,24], this is the only observation similar to our study. Both VD3-sufficient and VD3-deficient mice, in response to developed HP, revealed stimulation of breath frequency associated with lowered Ti and Total Cycle Time. On the contrary, Minute Volume, Time of expiratory, and marker for bronchoconstriction (EF50; mid-tidal expiratory flow) were altered only in animals on the restricted diet. Comparison data collected from animals on different diets revealed that VD3-deficiency significantly worsened the respiratory functions during HP development. All investigated parameters (F, MV, EF50, TCT, Ti, Te) in VD3-deficient mice were much more disturbed after 28 days of inhalations with *P. agglomerans* antigen than in VD3-sufficient mice. This observation leads to the reflection that an optimal cholecalciferol diet may mitigate the worsening of respiratory functions noted in HP, which, if confirmed in clinical studies, will beneficially impact the quality of patients’ lives. Unfortunately, there is a lack of data examining the effect of vitamin D supplementation on respiratory function, not only in HP but also in other pulmonary diseases, during which fibrosis is noted. Thus, it is worth mentioning a clinical trial that investigated the impact of co-supplementation with vitamins C, D, and E on, among others, respiratory responses in IPF patients. Participants in this study received vitamin E in a dose of 200 IU/day, 250 mg of vitamin C every other day for twelve weeks, and vitamin D3 in a dose of 50,000 IU/week for eight weeks. Collected data showed the beneficial impact of tested supplementation on IPF patients’ respiratory function, including enhancement of IRV (inspiratory reserve volume), FEV1 (forced expiratory volume in the first second), RV (residual volume), and TLC (total lung capacity). Nevertheless, there were no significant changes in VC (vital capacity), FVC (forced vital capacity), ERV (expiratory reserve volume), and FEV1/FVC in response to the treatment [29].

Histopathologic examination did not show any changes in the lung morphology of untreated mice dependent on vitamin D3 levels. Instead, flow cytometry showed a significant decrease in the amount of macrophages, neutrophils, and dendritic cells in VD3-deficient mice. Due to the important role of the indicated groups of immune cells in the body’s response to pathogens, the decrease in their number, along with the decrease in the concentration of the immune peptide CAMP and molecules integrating the cellular and humoral response (IL12 and IFNγ) recorded by ELISA in VD3-deficient mice, were a sign of their greater sensitivity to the development of HP. The disturbances in the immune response observed in healthy VD3-deficient mice do not fully correspond to the results of clinical studies, which revealed maturation disorders as well as a decrease in macrophage activity in the conditions of VD3 deficiency, but at the same time suggest that an insufficient amount of this metabolite leads to the stimulation of these processes in dendritic cells and their ability to produce IL12, as well as enhancing the Th1 response, including release of IL-2, IFN-γ, TNF-α [30,31,32]. As predicted, analysis of microscopic preparations taken from VD3-deficient mice revealed increased inflammation after 14 and 28 days of inhalation and signs of fibrosis after 28 days of antigen exposure. Although microscopically, VD3-deficient mice with HP had significantly more immunocompetent cells, flow cytometry showed that the most numerous cell sub-populations occurred in the lung homogenates from VD3-sufficient mice: neutrophils, macrophages, and dendritic cells after 14 days of antigen treatment, and in the case of M2 macrophages and dendritic cells also on day 28 of the experiment. In VD3-deficient mice, flow cytometry revealed increased amounts of lymphocytes Th1, Th2, and Tc after 14 days of inhalation with *P. agglomerans*, and neutrophils and lymphocytes Tc after 28 days of treatment. Unfortunately, there is a lack of research on the influence of vitamin D levels on the immune response in the course of HP that we could compare with our data. However, observed during histopathologic examination, the reduction of inflammatory infiltrates in VD3-sufficient mice with HP vs. VD3-deficient mice with HP corresponds to the data from the bleomycin-induced murine models of IPF treated with different forms of vitamin D3: cholecalciferol administered intraperitoneally [33], calcitriol administered through a tube [34], or intraperitoneally [35,36].

Interestingly, the cytokine profile showed that significant changes in the response of VD3-sufficient and VD3-deficient mice to *P. agglomerans* antigen were observed only on day 14 of the experiment in terms of pro-inflammatory IL6 and IL12 and the CAMP immune peptide. The concentration of these proteins was significantly higher in mice on the standard diet, and the obtained results were consistent with the previously mentioned higher levels of neutrophils, macrophages, and dendritic cells recorded in these animals. Interestingly, the observed changes in the levels of IL 6 and IL 12 are inconsistent with the above-mentioned results from mouse models of IPF treated with intraperitoneal calcitriol [35,36], but correspond to our earlier research, wherein VD3-deficient mice with HP were treated with inhaled calcidiol and calcitriol [18]. It is also worth noting that a comparison of data within dietary groups showed that the cytokine profile in VD3-sufficient mice remained virtually unchanged in response to HP-induction. There were only three statistically significant alterations in indicated research groups: on day 14, increased concentration of IL6 was observed, which correlated with an elevated influx of M2 macrophages and lymphocytes B; a decrease in IFNγ concentration associated with a lowered number of Th1 lymphocytes were also recorded on day 14 of inhalation; and the accelerated concentration of CAMP associated with an elevated amount of neutrophils in response to 28 days of antigen inhalation. A significant increase in the number of Treg lymphocytes, B lymphocytes, and antagonistic Th1 and Th2 lymphocytes may explain the stability of the cytokine profile in VD3-sufficient mice on day 28 of nebulization.

The cellular and cytokine profiles in VD3-deficient mice treated with antigen were difficult to interpret unambiguously. Inhalation of the antigen stimulated the influx of almost all investigated immune cells into the pulmonary parenchyma of VD3-deficient mice. Simultaneously, the concentrations of the majority of examined cytokines decreased in response to the antigen, except for CAMP and IFNγ. A statistically significant increase in IFNγ was recorded on day 28 of inhalation, while an elevated concentration of immune peptide was recorded after 14 days and 28 days of antigen treatment. Over-expression of CAMP seems to be connected with increased accumulation of neutrophils and macrophages in pulmonary compartments.

Alterations in the concentration of CAMP (cathelicidin antimicrobial peptide) in response to inhalations with HP-inducer, as well as different amounts of cholecalciferol in animals’ diets, are particularly interesting, since they prove that a VD3-standard diet can protect from the reduction in CAMP during HP development. Because of its antimicrobial, LPS-neutralizing, and wound-healing properties [37,38,39] as well as the negative correlation between cathelicidin concentration and tissue remodeling in lung fibrosis [40,41] and in advanced stages of COPD [42], maintenance of CAMP physiological concentration should be considered as a therapeutic strategy for pulmonary fibrosis. This is especially true since the results of our previous research confirmed the potential of such an approach [43,44].

Unfortunately, as previously mentioned, there is no data on the effect of vitamin D supplementation on the development of HP. When discussing the results of our team’s histological assessment and immunological analyses, it is worth mentioning the research conducted by Zhang et al. on mice with bleomycin-induced IPF treated with calcitriol administered at a dose of 0.5 µg/mL orally (by gavage). The indicated study provided a quite similar picture of the treatment effects to those presented herein. Mice with bleomycin-induced IPF showed thickening of alveolar septa, increased collagen deposition, and accumulation of inflammatory cells. Combined administration of bleomycin and calcitriol significantly attenuated these profibrotic changes. Furthermore, BALF analysis revealed a significant decrease in the total number of inflammatory cells, including alveolar macrophages, lymphocytes, and eosinophils, in the calcitriol-treated group compared to the bleomycin group. Moreover, the cytokine profile (IFNγ, IL4, IL12, IL13) was not statistically affected by the investigated calcitriol intervention [34].

As previously mentioned, histological analysis revealed focal atelectasis and marked thickening of the pulmonary septa and alveolar walls in mice after the 14th day of inhalation with *P. agglomerans* antigen. Development of pulmonary fibrosis was significantly exacerbated in VD3-deficient mice during the subsequent 14 days of antigen exposure. The disorders discovered in lung morphology corresponded with ELISA data, which revealed increased concentrations of fibrosis markers (collagen type 1 and hydroxyproline) and factors promoting this process (FGF2 and TGFβ). The ELISA results also explain the histologist’s observation of a greater susceptibility of VD3-deficient mice to fibrosis. Healthy VD3-deficient mice had higher concentrations of hydroxyproline, type 1 collagen, and FGF2, compared to the mice on a standard diet. This higher baseline amount of indicated proteins promoted the development of fibrosis in VD3-deficient animals. Furthermore, a comparison of data from VD3-sufficient and VD3-deficient mice with progressive HP showed significantly higher levels of fibrosis-promoting factors (FGF2 and TGFβ) in mice on the restricted diet, which also explains the greater susceptibility of these animals to fibrosis induction. Because of the proven pivotal role of TGFβ and FGF2 in fibrosis development and reports of antifibrotic therapies based on inhibiting the levels of these proteins [45,46,47,48,49], the discovery of the beneficial impact of a VD3-sufficient diet on the expression of indicated growth factors suggests the possibility of using VD3 supplements as cheap and safe modulators of these molecules. Nevertheless, taking into account the decreasing level of the bioactive form of vitamin D3 (calcitriol) during the development of pulmonary fibrosis, even in animals on a diet with the recommended content of cholecalciferol, basing HP therapy on vitamin D3 supplementation must include this observation.

The increased concentrations of fibrosis markers (hydroxyproline and collagen type 1) observed during HP development, and above all, factors promoting pathological tissue repair (FGF2 and TGFβ), the levels of which were inversely dependent on the pulmonary calcitriol concentration, prompted us to take a closer look at the vitamin D3 impact on the phenomenon of epithelial-mesenchymal transition (EMT), which underlies tissue fibrosis [50,51]. The EMT consists of a loss of integrity of normal epithelium, including loss of their polarity and decline of cell-to-cell adhesion molecules, as well as acquisition of the mesenchymal phenotype, characterized by elongated morphology, increased migratory capacity, and enhanced extracellular matrix deposition. It is worth explaining that there are three different types of EMT, among which type 2 occurs in tissue regeneration and organ fibrosis. This type of EMT is strongly related to inflammation, and attenuated in response to completion of physiological repair, which stops EMT. In the case of fibrosis, the EMT type 2 and inflammation drive each other until the fibrotic process reaches the point where it can no longer be stopped by suppressing inflammation [50,51,52]. Our research team was the first to prove the involvement of EMT type 2 in pulmonary fibrosis related to HP, showing down-regulation of epithelial markers (*Cdh1*/E-cadherin, *Ocln*/Occludin, *Cldn1*, *Jup*) with simultaneous up-regulation of mesenchymal markers (*Acta2*/α-SMA, *Cdh2*/N-cadherin, *Fn1*/Fibronectin, *Vim*/Vimentin) supported by elevated levels of EMT-transcription factors (*Snai1*/Snail, *Snail2*, *Zeb1*/ZEB1, *Zeb2*/ZEB2, *Nfkb*/NFκB, *Tgfb1*/TGFβ, *Ctnnd1*/β-catenin) [53,54]. Unfortunately, there is no data describing the influence of dietary cholecalciferol levels on EMT in pulmonary fibrosis in the course of HP. The present study fills in this knowledge gap.

At the beginning of this part of the study, we focused on the expression of four transcription factors—members of the *Snail* and *Zeb* families—with pivotal roles in the EMT as well as increased deposition of extracellular matrix components and their further rearrangement [51,55,56]. Our studies revealed for the first time that healthy VD3-deficient mice have significantly higher expression levels of EMT-transcription factors [50,51], i.e., *Snail2*, *Zeb1* and *Zeb2*, than VD3-sufficient mice. As expected, as *P. agglomerans* antigen-induced pulmonary fibrosis progressed, expression of *Snail2*, *Zeb1*, and *Zeb2* increased in both VD3-sufficient and VD3-deficient mice, but their levels were significantly higher in animals on the restricted diet. Moreover, in VD3-sufficient mice, the expression of *Snail1* intensified with the time of antigen exposure, whereas in VD3-deficient mice, an increased level of this factor was observed only on the 28th day of inhalation. The discovered silencing impact of vitamin D3 on the expression of EMT-transcription factors corresponds to results of our previous investigation, which revealed significant down-regulation of *Snail1*, *Snail2*, *Zeb1*, and *Zeb2* in VD3-deficient mice with HP inhaled with calcidiol or calcitriol [19]. Furthermore, the presented results correspond with data collected from in vitro studies, wherein calcitriol inhibits EMT induced by TGFβ, among others, through modulation of expression of Snail in rat alveolar epithelial cells [57], human bronchial epithelial cells [58], and human lung cancer A549 cells—a model of type II alveolar epithelial cells [59]. Unfortunately, there is a lack of in vivo or clinical data investigating the influence of vitamin D supplementation on EMT-transcription factors. Nevertheless, the discovered beneficial impact of a cholecalciferol-standard diet on the expression of the most important EMT-transcription factors suggested that maintaining the physiological level of calcitriol through an appropriate diet may be the key to preventing HP-related pulmonary fibrosis by suppressing the pathological variant of EMT type 2. VD3 supplementation may prove to be as effective a therapeutic option as previously and currently investigated silencing expression of members of the Snail and Zeb families, e.g., Nrf2 (nuclear erythroid 2 related factor), which inhibits the pulmonary fibrosis process by down-regulation of *Snail* [60]; miR-200b/c, which targets both ZEB1 and ZEB2, attenuates lipopolysaccharide-induced early pulmonary fibrosis [61], or proposed by Cai et al. “MSI2–ZEB1 axis inhibition” protects from radiation-induced pulmonary fibrosis [62].

Further studies on the effect of dietary vitamin D3 levels on the course of EMT focused on the expression of epithelial (*Cdh1*/E-cadherin, *Ocln*/Occludin) and mesenchymal (*Cdh2*/N-cadherin, *Fn1*/Fibronectin, *Acta2*/α-SMA, *Vim*/Vimentin) cell markers, which are considered as reliable biomarkers of fibrosis progression [51,55,56]. Studies conducted at both the mRNA and protein levels showed that in healthy animals, the expression levels of all the molecules indicated above were similar in both dietary groups. As expected, during HP progression, the expression of epithelial markers (*Cdh1*, *Ocln*) decreased, and the expression of mesenchymal cell markers (*Cdh2*/N-cadherin, *Fn1*/Fibronectin, *Acta2*/α-SMA, *Vim*/Vimentin) increased. In the case of indicated RT-PCR results, the described changes increased with exposure time, but microscopic analysis did not confirm this trend. Nevertheless, it is worth emphasizing that both gene expression assays and immunohistochemistry revealed that VD3-deficiency intensified the expression of mesenchymal markers compared to data collected from VD3-sufficient animals. Furthermore, this observation corresponds with the previously described histological results, showing significantly higher signs of fibrosis at the end of the study in VD3-deficient mice than in animals on a standard diet. The described beneficial effect of a diet rich in cholecalciferol on the expression level of EMT markers is consistent with the observations of other scientists who, in a murine model of bleomycin-induced IPF, showed that the administration of cholecalciferol reduced the expression of *Acta2*/α-SMA [33,63], a similar effect was noted after the administration of calcitriol [36], and paricalcitol (synthetic analogue of vitamin D3), which additionally reduced the expression of *Fn1*/Fibronectin [64].

Nevertheless, among the cited research, only Tziales et al. administered vitamin D (cholecalciferol, 2 µg/kg, for 10 days) [63] orally, while in the remaining studies, vitamin D metabolites were injected intraperitoneally [33,36,64]. The presented data also correspond with the results of our previous studies, which proved EMT inhibition in VD3-deficient mice with HP inhaled with calcidiol and calcitriol. Investigated metabolites up-regulated the expression of *Cdh1*/E-cadherin and *Olcn*, and simultaneously down-regulated the expression of *Acta2*, *Cdh2*, *Fn1*/Fibronectin, and *Vim*/Vimentin. On the contrary, expression of Occludin and N-cadherin in the case of calcitriol treatment, as well as α-SMA, revealed the opposite effect from the data presented [19]. Next to the above-mentioned results of in vivo research, there is a much larger panel of in vitro studies in which exposure of lung cells to vitamin D inhibited TGFβ-induced EMT by modulating the expression of epithelial and mesenchymal cell markers. Among the indicated studies worth mentioning are those conducted on pulmonary epithelial cells. Zheng et al. conducted studies on human primary alveolar type II cells that have shown that both calcidiol and calcitriol, administered together with TGFβ, increased the expression of *Cdh1*/E-cadherin and, at the same time, decreased the expression of *Cdh2*/N-cadherin, *Acta2*/α-SMA and *Vim* [65]. Fischer and Agrawal investigated human bronchial epithelial cells treated with TGFβ and calcitriol and observed elevated levels of *Cdh1*/E-cadherin, as well as lowered levels of *Cdh2*/N-cadherin and *Vim*/Vimentin [58]. Similarly, Chen et al. 2016 investigated calcitriol impact on rat alveolar epithelial cells exposed to TGFβ and recorded an increased amount of E-cadherin as well as down-regulated expression of Fibronectin and α-SMA [66].

## 4. Materials and Methods

### 4.1. Reagents

Reagents were obtained from Sigma-Aldrich Co. LLC (Burlington, MA, USA), unless otherwise stated. The preparation of *P. agglomerans* antigen has been described previously [25,44].

### 4.2. Research Design

Research was carried out on three-month-old male C57BL/6 mice from the Mossakowski Medical Research Centre of the Polish Academy of Sciences in Warsaw, Poland (36 animals; mean weight 24.5 g). Animal studies were performed in the animal facility of the Institute of Rural Health in Lublin, Poland. After delivery, animals were randomly assigned to research groups (6 mice/group). Animals were kept in separate polycarbonate cages under the following conditions: natural light–dark cycle temperature 20–24 °C, air humidity 45–65%, 15 air changes per hour, unlimited access to fresh water and food. Vitamin D3-sufficient mice (VD3S) received a diet with a standard cholecalciferol level (0.5 IU/g), while vitamin D3-deficient mice (VD3D) were provided with a diet with 10-times-less cholecalciferol (0.05 IU/g), purchased from Altromin (Altromin Spezialfutter GmbH & Co. KG, Lage, Germany). After delivery, the animals underwent a week of acclimatization, followed by a subsequent week of adaptation to the inhalation chambers and accessories (neck restrainers and nose-mouth silicon masks). The actual research began just after the indicated acclimatization and adaptation procedures. Vitamin D3 statuses (VD3S and VD3D) were confirmed by examination of pulmonary and serum levels of calcitriol (bioactive form of vitamin D3) performed according to procedures presented previously [18,24]. In order to induce hypersensitivity pneumonitis, mice were exposed to the antigen of *P. agglomerans* at doses of 5 mg/mouse. The antigen was delivered directly to the respiratory tracts of the mice via nebulization, using the Buxco Inhalation Tower (Data Sciences International, St. Paul, MN, USA) under the following conditions: airflow 2.5 L/min, pressure −0.5 cm H_2_O, room temperature, average nebulization rate 348 μL/min. Nebulization was carried out for thirty minutes daily for 14 days or 28 days.

**Figure 8:**
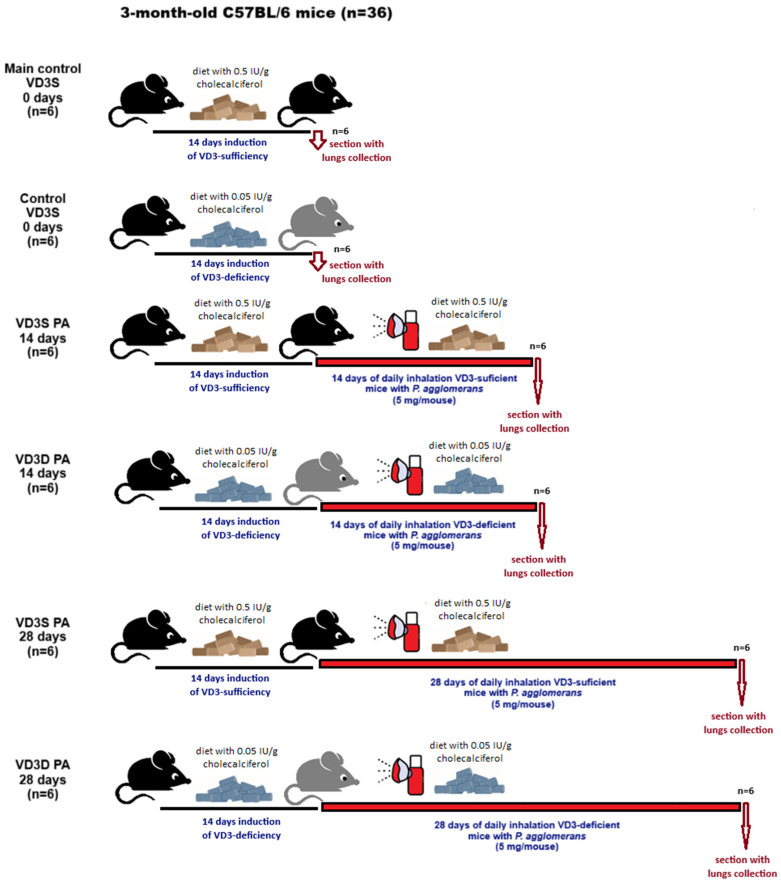
Research plan.

The final step of every single experiment was whole-body plethysmography. The mice were placed in a non-invasive airway mechanics plethysmograph chamber (Data Sciences International, St. Paul, MN, USA). Respiratory parameters, including the frequency of breathing (F), minute volume (MV), inspiratory time (Ti), expiratory time (Te), mid-tidal expiratory flow (EF50), and total cycle time (TCT), were determined using DSI FinePointe Software v 2.3.1.21 under the following conditions: airflow 0.6 L/min; pressure 0 cm H_2_O; room temperature. Each experiment ended with the humane killing of the mice via cervical dislocation with spinal cord rupture, followed by dissection and collection of blood and lungs for further studies. The graphical summary of the research design is presented in Figure 8. The research protocols were approved by the Local Ethics Committee for Animal Experimentation in Lublin, Poland (Resolution Nos. 28/2021, 25/2022, and 73/2022).

### 4.3. Determination of Calcitriol Concentration—ELISA Method

A detailed description of both blood and lung tissue sample preparation for examination of calcitriol concentration has been presented previously [24]. The serum level of calcitriol was assessed using the Mouse 1,25-dihydroxyvitamin D3 (DVD/DHVD3) ELISA (Shanghai Coon Koon Biotech Co., Ltd., Shanghai, China), following the manufacturer’s instructions. The pulmonary level of calcitriol was evaluated using the 1,25(OH)2 vitamin D Total ELISA (BioVendor R&D, Brno, Czech Republic), according to the manufacturer’s instructions. Data obtained from the lung tissue were normalized to the total protein concentration in the tested homogenates, as previously described [24].

### 4.4. Determination of Immune Cells Composition—Flow Cytometry

Lung tissue preparation for flow cytometry determination has been presented in detail previously [18]. To determine the phenotype of immune cells isolated from the lung homogenates, the following markers were used: dendritic cells (CD103, CD11b, CD209), lymphocytes B (B220, CD19), lymphocytes Tc (CD3, CD8), lymphocytes Th1 (CD3, CD4, IFNγ), lymphocytes Th2 (CD3, CD4, IL4), lymphocytes Treg (CD3, CD4, CD25, FoxP3), M1 macrophages (CD11b, F4/80, CD86), M2 macrophages (CD11b, F4/80, CD206), and neutrophils (CD11b, Ly-6G/Gr-1). Antibodies and corresponding isotype controls were purchased from BD Biosciences (San Diego, CA, USA), with the exception of antibodies against CD206 and its isotype control, which were obtained from Life Technologies Corporation (Carlsbad, CA, USA). A detailed description of immune cell staining has been presented previously [18]. Stained cells were investigated using a BD Accuri C6 Plus flow cytometer with BD CSampler Plus software v.1.0.34 (BD Biosciences, San Jose, CA, USA). The gating strategies for both surface and intracellular staining are presented in the Supplementary Materials Figure S1.

### 4.5. Evaluation of Lung Tissue Injury Scores—Masson Trichrome Staining

Lungs were fixed in 4% buffered formalin. After dehydration, tissue samples were embedded in paraffin wax and then cut into 3 μm sections and stained with Masson trichrome. Then, stained tissues were assessed in an MW50 light microscope (OPTA-TECH, Warsaw, Poland) by an experienced histopathologist for changes in morphology and signs of inflammation and fibrosis. Lung injury was graded with the 5-point Murray’s scale: regular tissue = 0 points; slight injury to 25% = 1 point; moderate injury to 50% = 2 points; severe injury to 75% = 3 points; very severe injury to around 100% = 4 points. The indicated parameters were analyzed twice, in the entire stained slide, and the final visual scores were calculated as the median of the investigated items in each research group. To record observed changes, micrographs were prepared in Capture v2.0 software (OPTATECH, Warsaw, Poland).

### 4.6. Visualization of Selected Protein Expression-Immunohistochemistry

Evaluation of EMT protein expression was performed on FFPE tissue cut into 3 μm sections and fixed on Superfrost Plus Microscope Glass Slides (Thermo Scientific, Brunschweig, Germany). Before immunohistochemistry, slides with lung sections were preheated at 59 °C on a hotplate for at least three hours. Staining of EMT-markers was performed using antibodies that specifically detected N-Cadherin, Fibronectin, Occludin, E-Cadherin, Vimentin, and α-SMA, according to the manufacturer’s instructions (abcam, Cambridge, UK). To visualize the antigen-antibody reaction, the appropriate Deko Real EnVision/HRP (DAB+) detection systems for mouse and rabbit primary antibodies were used. After a 30 min incubation of the sections with a secondary antibody labeled with horseradish peroxidase, an enzymatic reaction was performed using the peroxidase substrate 3,3-diaminobenzidine tetrachloride (DAB), according to the manufacturer’s instructions (Agilent Technologies, Santa Clara, CA, USA). After staining, the microscope slides were washed and dehydrated in a series of two 96% ethanol and two xylene washing steps, and then cover-slipped. Alterations in the expression of epithelial and mesenchymal cell markers were examined under an MW50 light microscope (OPTA-TECH, Warsaw, Poland) using the 5-point scale: no reaction/expression among target cells = 0 points; 1–25% of target cell with positive reaction = 1 point; 26–50% = 2 points; 51–75% = 3 points; >75% = 4 points). The assessment of protein expression was performed on the entire stained slide, and the final visual scores from two examinations were calculated as the median of the investigated items in each research group. Changes in the expression of EMT-markers were visualized using an MW50 light microscope with Capture v2.0 software (OPTA-TECH, Warsaw, Poland).

### 4.7. Evaluation of Gene Expression—Real Time PCR

Isolation and purification of nucleic acids for gene expression assay have been presented previously [19]. Obtained RNA (500 ng) was reverse-transcribed using a High Capacity cDNA Reverse Transcription Kit (ThermoFisher Scientific, Vilnius, Lithuania). Real-time PCR was then conducted using TaqMan Fast Universal PCR MasterMix and TaqMan Gene Expression Assays designed to specifically recognize the following mouse genes: *Acta2*, *Actb*, *Cdh1*, *Cdh2*, *Fn1*, *Ocln*, *Snail1*, *Snail2*, *Vim*, *Zeb1*, *Zeb2* (Applied Biosystems, Foster City, CA, USA). The details of the probe and primer sets and reaction conditions were presented earlier [19]. Gene expression assays were performed using a 7500 Fast Real-Time PCR System with SDS 1.4 Software (Applied Biosystems, Waltham, MA, USA). Relative gene expressions were calculated using the relative advanced quantification and normalized to the expression of *Actb*.

### 4.8. Examination of Selected Protein Concentration—ELISA Method

The preparation of lung tissue homogenates for protein determination has been previously presented in detail [44]. Protein concentrations were estimated using ELISA Kits designed to specifically recognize the following mouse proteins: Cathelicidin, Collagen type 1, FGF2, Hydroxyproline, TGFβ, IFNγ, IL1β, IL4, IL6, IL10, IL12, and IL13 (Cloud-Clone Corp., Katy, TX, USA).

### 4.9. Statistical Analysis

Statistical analysis aimed at identifying differences between the research groups was conducted utilizing the Wilcoxon–Mann–Whitney test, a non-parametric approach. To address the challenge of multiple comparisons, the Benjamini–Hochberg procedure of controlling the false discovery rate (FDR) was also applied. Statistical significance was defined by a corrected *p*-value of less than 0.05. The magnitude of the difference separating the groups was quantified using the Hodges–Lehmann estimator. This provides the median of all possible pairwise differences among the observations within the groups being compared. To estimate the probability that one observation exceeds the other (P(X > Y)), the statistical power of the Wilcoxon–Mann–Whitney test was evaluated. This evaluation was performed using the shiehpow function from the wmwpow R package (version 0.1.3). Relationships between variables were assessed via Spearman’s rank correlation coefficient. All statistical computations and data visualizations were performed in the R environment (version 4.5.0.), specifically employing the ggpubr (version 0.6.0) and ggplot2 (version 3.5.2.) software packages. In the accompanying figures, data are displayed as box plots. The median and the interquartile range (IQR) are marked by the central line and the box edges, respectively, while the whiskers visualize the minimum and maximum observed values. Three distinct reference schemes were employed for the statistical comparisons depicted in the box plots. Each scheme was clearly specified by a unique symbol within the figure legends. Specifically, the schemes compared the experimental groups with the following: (1) the main control group (VD3S 0 days; *), (2) the baseline for the vitamin D-deficient group (VD3D 0 days; ^), and (3) the vitamin D-sufficient group at matched time points (VD3S PA 14/28 days; #). Consistent across all figures, statistical significance is denoted as follows: a single symbol (e.g., *) denotes *p* < 0.05, a double symbol (e.g., **) denotes *p* < 0.01, and a triple symbol (e.g., ***) denotes *p* < 0.001.

## 5. Conclusions

To the best of our knowledge, the present study is the first one to investigate the impact of the amount of cholecalciferol in the diet on the development of pulmonary fibrosis in the course of HP. The performed research demonstrated that VD3-deficiency promotes fibrosis development in response to chronic exposure to the antigen of *P. agglomerans*, thus worsening the respiratory functions, including frequency of breathing, TCT, Te, Ti, MV, and EF50. Enhancement of fibrosis development in VD3-deficient mice with developing HP was strongly associated with an increased influx of immune cells into the lungs (especially neutrophils, macrophages, dendritic cells and lymphocytes Tc), disturbed release of several cytokines (lowered amount of IL1β, IL6, IL12, IL4 IL10, IL13), intensified production of growth factors favouring fibrosis response (FGF2 and TGFβ), and, most important of all, accelerated EMT underlying fibrosis (over-expression of EMT-transcription factors and mesenchymal cell markers). The indicated negative changes recorded in VD3-deficient mice with HP were supported by deepening calcitriol deficiency. The presented results bring hope for the elaboration of an easy, cheap, and safe prevention strategy for HP-related pulmonary fibrosis based on vitamin D supplementation. Nevertheless, because of the decrease in calcitriol levels accompanying the HP development observed even in VD3-sufficient diets, basing a fibrosis prevention strategy on vitamin D supplementation must take into account the discovered negative changes in this hormone resource.

## Figures and Tables

**Table 1:** Pulmonary and serum calcitriol (1,25(OH)_2_-VD3) concentrations following *P. agglomerans* inhalation and/or vitamin D3 deficiency. Data represent median values determined by ELISA. Statistical significance was assessed using the Wilcoxon–Mann–Whitney test with Benjamini–Hochberg correction. Symbols denote specific comparisons: (*) vs. main control (VD3S 0 days); (^) vs. VD3D baseline (0 days); and (#) VD3D vs. VD3S at matched time points (14 or 28 days). The number of symbols indicates the significance level: two (*p* < 0.01), or three (*p* < 0.001). The table contains some data, which have been presented previously [18,19].

	VD3S 0 Days	VD3D 0 Days	VD3S PA 14 Days	VD3D PA 14 Days	VD3S PA 28 Days	VD3D PA 28 Days
pulmonary level of 1,25(OH)2-VD3 [pg/mg]	30.21	*** 18.21	*** 27.89	***, ^^, ### 15.83	*** 24.07	***, ^^^, ### 13.30
serum level of 1,25(OH)2-VD3 [pg/mL]	132.71	*** 98.63	*** 116.73	***, ^^^, ### 78.68	*** 119.34	***, ^^^, ### 69.86

## Data Availability

The original contributions presented in this study are included in the article/Supplementary Material. Further inquiries can be directed to the corresponding author.

## Supplementary Materials

The following supporting information can be downloaded at https://www.mdpi.com/article/10.3390/ijms262411770/s1.
