# Supplementary material for: Vitamin D3-Deficient Diet Promotes Pulmonary Fibrosis Development in Murine Model of Hypersensitivity Pneumonitis
Source: Int J Mol Sci. 2025 Dec 5;26(24):11770. doi: 10.3390/ijms262411770 (PMC12733112; doi:10.3390/ijms262411770)
Supplement: Supplementary file 1 [file ijms-26-11770-s001.zip › Table S1.pdf]

Table S1. Alterations in pulmonary function caused by vitamin D3 deficiency and chronic exposure to antigen of *Pantoea agglomerans*. Respiratory parameters were investigated using the Whole Body Plethysmography. Data are presented as median.

	**VD3S 0 days**	**VD3D 0 days**	**VD3S PA 14 days**	**VD3D PA 14 days**	**VD3S PA 28 days**	**VD3D PA 28 days**
**Frequency of breathing [breaths/min]**	261.87	291.75	333.89	363.22	331.04	399.64
**Minute Volume [mL/min]**	82.74	96.18	88.14	71.99	87.14	143.81
**EF50 [mL/s]**	3.46	3.99	4.04	3.61	3.56	6.15
**Total Cycle Time [s]**	0.24	0.22	0.20	0.18	0.19	0.15
**Time of inspiratory [s]**	0.10	0.09	0.07	0.07	0.08	0.07
**Time of expiratory [s]**	0.14	0.13	0.12	0.11	0.12	0.08
