# Supplementary material for: Vitamin D3-Deficient Diet Promotes Pulmonary Fibrosis Development in Murine Model of Hypersensitivity Pneumonitis
Source: Int J Mol Sci. 2025 Dec 5;26(24):11770. doi: 10.3390/ijms262411770 (PMC12733112; doi:10.3390/ijms262411770)
Supplement: Supplementary file 1 [file ijms-26-11770-s001.zip › Table S2.pdf]

Table S2. Changes in pulmonary composition of immune cells in response to vitamin D3 deficiency and chronic exposure to antigen of *Pantoea agglomerans*. Flow cytometry data are presented as a median of percentage of all living immune cells

	**VD3S 0 days**	**VD3D 0 days**	**VD3S PA 14 days**	**VD3D PA 14 days**	**VD3S PA 28 days**	**VD3D PA 28 days**
**neutrophils CD11b+ Ly-6G+**	6.27	3.71	26.35	16.37	21.97	34.47
**macrophages M1 CD11b+ F4/80+ CD86+**	7.93	0.53	7.97	4.23	7.63	10.09
**macrophages M2 CD11b+ F4/80+ CD206+**	8.01	2.89	11.19	6.41	10.23	2.79
**dendritic cells CD11b+ CD103+ CD209+**	6.05	2.90	7.63	13.72	5.80	9.20
**lymphocytes B B220+ CD19+**	0.36	0.19	4.88	1.01	4.89	0.87
**lymphocytes Tc CD3+ CD8+**	6.64	4.24	3.38	6.15	6.35	9.36
**lymphocytes Th1 CD3+ CD4+ IFN-gamma+**	0.37	0.21	0.08	0.58	2.67	0.57
**lymphocytes Th2 CD3+ CD8+ IL-4+**	0.06	0.08	0.02	0.46	1.21	0.57
**lymphocytes Treg CD4+ CD25+ FOXP3+**	0.02	0.02	0.87	0.31	0.25	0.46
