# Supplementary material for: Vitamin D3-Deficient Diet Promotes Pulmonary Fibrosis Development in Murine Model of Hypersensitivity Pneumonitis
Source: Int J Mol Sci. 2025 Dec 5;26(24):11770. doi: 10.3390/ijms262411770 (PMC12733112; doi:10.3390/ijms262411770)
Supplement: Supplementary file 1 [file ijms-26-11770-s001.zip › Table S3.pdf]

Table S3. Changes in the cytokines and CAMP concentration caused by to vitamin D3 deficiency and chronic exposure to antigen of *Pantoea agglomerans*. Protein concentration was determined in lung tissue homogenates using ELISA. Data are presented as median.

	**VD3S 0 days**	**VD3D 0 days**	**VD3S PA 14 days**	**VD3D PA 14 days**	**VD3S PA 28 days**	**VD3D PA 28 days**
**IL1β [pg/mL]**	279.16	542.46	358.39	300.65	292.20	250.88
**IL6 [pg/mL]**	296.27	387.18	396.11	231.17	238.77	263.67
**IL12A [pg/mL]**	177.96	105.46	184.12	66.41	183.31	154.35
**IFNγ [pg/mL]**	840.42	372.47	565.27	220.55	929.83	868.71
**IL4 [pg/mL]**	371.02	396.41	329.53	306.51	263.02	297.51
**IL10 [pg/mL]**	192.35	211.15	144.80	136.95	138.06	137.51
**IL13 [pg/mL]**	1273.39	1454.78	1119.51	982.77	769.74	1273.39
**CAMP [ng/mL]**	391.51	211.27	412.22	339.57	444.65	271.28
