# Supplementary material for: Vitamin D3-Deficient Diet Promotes Pulmonary Fibrosis Development in Murine Model of Hypersensitivity Pneumonitis
Source: Int J Mol Sci. 2025 Dec 5;26(24):11770. doi: 10.3390/ijms262411770 (PMC12733112; doi:10.3390/ijms262411770)
Supplement: Supplementary file 1 [file ijms-26-11770-s001.zip › Table S4.pdf]

Table S4. Alterations in extracellular matrix deposition in response to vitamin D3 deficiency and chronic exposure to antigen of *Pantoea agglomerans*. Protein concentration was determined in homogenates of lung tissue using ELISA Kits. Data are presented as medians.

	**VD3S 0 days**	**VD3D 0 days**	**VD3S PA 14 days**	**VD3D PA 14 days**	**VD3S PA 28 days**	**VD3D PA 28 days**
**Hydroxyproline [ng/mL]**	3.92	7.40	14.49	16.10	16.99	18.43
**Collagen type 1 [pg/mL]**	1252.67	1561.80	1733.79	1724.03	1411.90	1758.52
**FGF2 [pg/mL]**	32.64	74.67	69.39	101.83	85.63	135.74
**TGFβ [pg/mL]**	346.53	394.52	475.29	674.64	502.78	695.23
