# Supplementary material for: Vitamin D3-Deficient Diet Promotes Pulmonary Fibrosis Development in Murine Model of Hypersensitivity Pneumonitis
Source: Int J Mol Sci. 2025 Dec 5;26(24):11770. doi: 10.3390/ijms262411770 (PMC12733112; doi:10.3390/ijms262411770)
Supplement: Supplementary file 1 [file ijms-26-11770-s001.zip › Table S5.pdf]

Table S5. Changes in the gene expression of EMT markers in response to vitamin D3 deficiency and chronic exposure to antigen of *Pantoea agglomerans*. Gene expression was investigated in lung homogenates using real-time PCR methods. Data are presented as median.

	**VD3S 0 days**	**VD3D 0 days**	**VD3S PA 14 days**	**VD3D PA 14 days**	**VD3S PA 28 days**	**VD3D PA 28 days**
Snail1	1.02	1.11	1.14	1.24	1.21	1.39
Zeb1	0.99	1.34	1.26	1.51	1.49	2.10
Snail2	1.03	1.39	1.10	1.51	1.46	2.08
Zeb2	1.02	1.35	1.24	1.77	1.40	1.86
Cdh1	1.03	1.00	0.70	0.57	0.86	0.77
Cdh2	1.00	1.11	1.24	1.45	1.47	1.48
Acta2	1.04	0.91	1.14	1.60	1.35	2.23
Ocln	1.00	0.96	0.55	0.45	0.64	0.42
Fn1	0.98	1.08	1.64	1.94	1.82	2.49
Vim	1.01	1.08	1.32	1.57	1.22	1.59
