# Supplementary material for: Vitamin D3-Deficient Diet Promotes Pulmonary Fibrosis Development in Murine Model of Hypersensitivity Pneumonitis
Source: Int J Mol Sci. 2025 Dec 5;26(24):11770. doi: 10.3390/ijms262411770 (PMC12733112; doi:10.3390/ijms262411770)
Supplement: Supplementary file 1 [file ijms-26-11770-s001.zip › Table S6.pdf]

Table S6. Alterations in the expression of EMT markers in response to vitamin D3 deficiency and chronic exposure to antigen of *Pantoea agglomerans*. Results obtained from visual quantification of protein expression on immunohistochemically stained lung tissue are presented as median.

	**VD3S 0 days**	**VD3D 0 days**	**VD3S PA 14 days**	**VD3D PA 14 days**	**VD3S PA 28 days**	**VD3D PA 28 days**
**E-cadherin**	1	1	1,5	1	1	1
**N-cadherin**	1	1	1,5	3	2	2
**Fibronectin**	1	1,5	1	2	1	2
**Occludin**	1	1	2	1	1	1
**α-SMA**	0	0,5	1,5	2	1	2
**Vimentin**	1	0,5	1	2	1	2
