# Supplementary figures and images for: Vitamin D3-Deficient Diet Promotes Pulmonary Fibrosis Development in Murine Model of Hypersensitivity Pneumonitis
Source: Int J Mol Sci. 2025 Dec 5;26(24):11770. doi: 10.3390/ijms262411770 (PMC12733112; doi:10.3390/ijms262411770)

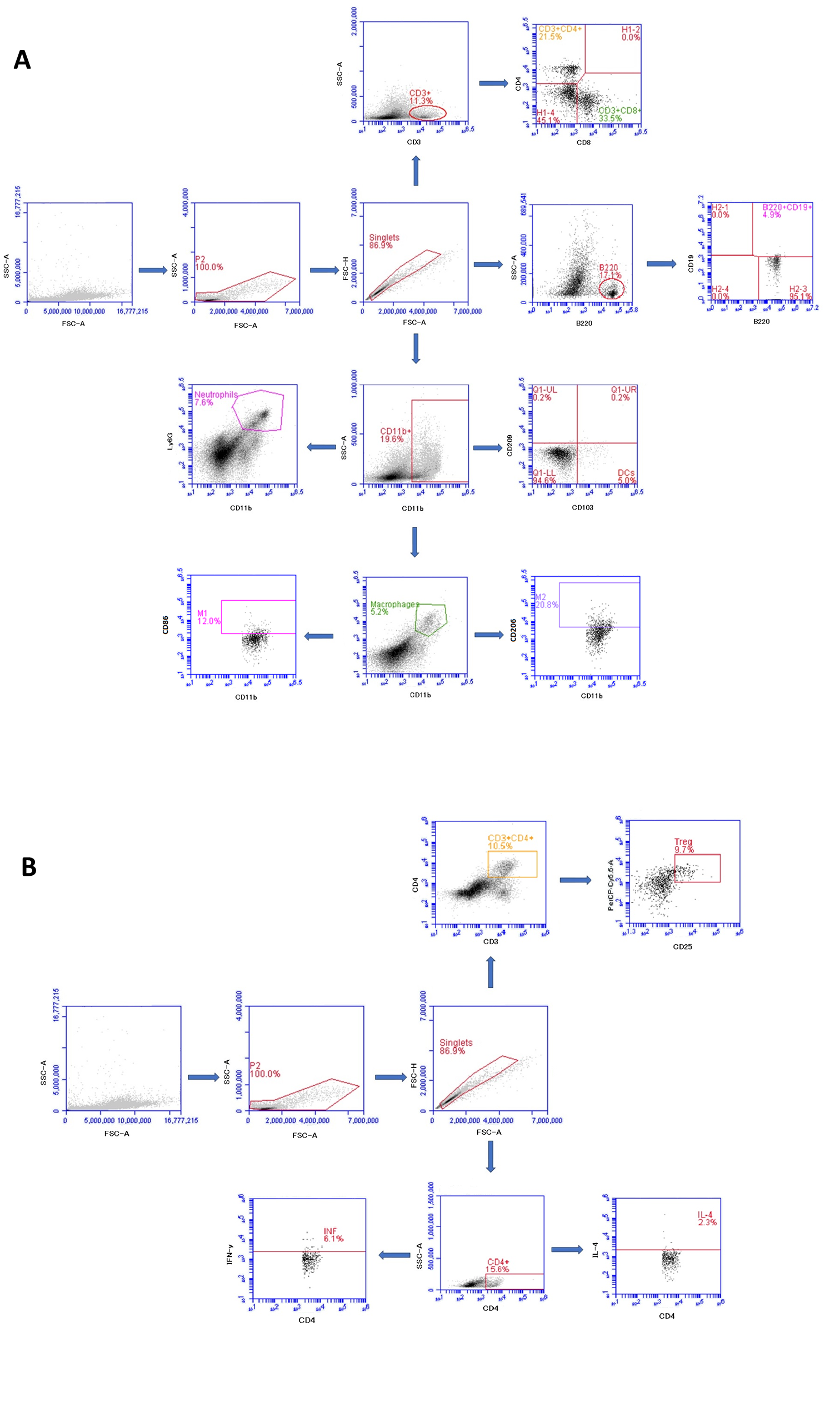

Supplement: Supplementary file 1 [file ijms-26-11770-s001.zip › Figure S1. The gating strategies for surface and intracellular staining.tif]
